# Patient and staff experiences of quality in Swedish forensic psychiatric care: a repeated cross-sectional survey with yearly sampling at two clinics

**DOI:** 10.1186/s13033-019-0265-z

**Published:** 2019-02-02

**Authors:** Mikael Selvin, Kjerstin Almqvist, Lars Kjellin, Lars-Olov Lundqvist, Agneta Schröder

**Affiliations:** 1 University Health Care Research Center, Faculty of Medicine and Health, Örebro University, 701 85 Örebro, Sweden; 2 Department for Social and Psychological studies, Karlstad University, Karlstad, Sweden; 3 Department of Nursing, Faculty of Health, Care and Nursing, Norwegian University of Science and Technology (NTNU), Gjövik, Norway

**Keywords:** Forensic psychiatry, Forensic nursing, Quality improvement

## Abstract

**Background:**

Systematic efforts to improve the quality, safety and value of health care have increased over the last decades. Even so, it is hard to choose priorities and to know when the desired results are reached, especially in forensic psychiatric care where there can be a discrepancy between patient and staff expectations of what good quality of care is and how it should be reached. The aim of the present study was to describe and compare patient and staff experiences of quality of care in two forensic psychiatric clinics over a period of 4 years.

**Methods:**

A quantitative design was used and yearly between 2011 and 2014, a total of 105 questionnaires were answered by patients and 598 by staff.

**Results:**

The sample consisted of four different groups; patient and staff in Clinic A and Clinic B respectively. The repeated measurements showed that quality of care, as described by the patients, varied over time, with significant changes over the 4 years. The staff evaluations of the quality of care were more stable over time in both clinics compared with the patients. Generally, the staff rated the quality as being better than the patients but these differences tended to decrease when efforts were made to improve the care.

**Conclusions:**

It is important to highlight both what staff and patients perceive as both high and low quality care. With regular measurements and sufficient resources, training, support and leadership, the chances of successful improvement work increase. This knowledge is important in forensic nursing practice, for teaching and for management and decision makers in the constant work of improving forensic psychiatric care.

## Background

Forensic psychiatry is a specialised field that involves the care and treatment of offenders with mental disorders and others requiring similar services [1]. Patients in forensic psychiatric care are involuntarily admitted which means a limitation in autonomy, and they often have complex circumstances which can include psychosocial, economic, environmental, and drug related problems in addition to the psychiatric disorder(s) [2]. This might lead to challenges for staff members when developing a care-promoting climate of high quality, since they have to deal with illness, crime and security issues, while simultaneously treating and attempting to improve the patients’ mental health [3, 4]. A high standard care in forensic psychiatry is essential, and if effective could also lead to reduced risk of violence [5].

The knowledge of systematic efforts to improve the quality, safety and value of health care has grown over the last decades [6, 7]. Worldwide, the education of health professionals now includes improvement as a standard competency [8–10]. Generally, a health care provider should maintain, but also constantly improve care so that it is effective, efficient, accessible, patient-centred, equitable and safe [11]. However, like with all improvement work, it may be difficult to make the right priorities and to know when the desired results are achieved [12]. This is especially true in forensic psychiatric care, where there can be a discrepancy between patient and staff expectations of what good quality of care is and how it should be reached [13–15]. Even if there are many similarities in what we mean with the concept quality of care, it is also known that differences exist, depending on which perspective we have [16]. It can be argued though, that this is not necessarily a problem because being aware of these discrepancies could lead to better understanding [17]. Therefore, it is necessary to integrate both patient and staff views in any evaluation of the care [18].

In order to systematically improve healthcare; it is important to have systems that continuously monitor and measure the care in a reliable manner [19]. There are, however, few studies that explore how patients and staff simultaneously perceive the quality of forensic psychiatric care. One reason for this is that there has been a lack of validated measurement instruments which cover the same dimensions of quality of care for use with both patients and staff. Therefore, two similar instruments; QPC-Forensic-In-Patient (QPC-FIP) and QPC-Forensic-Inpatient-Staff (QPC-FIPS) [20, 21] have been developed from the original version of Quality in Psychiatric Care (QPC) [22, 23]. These instruments measure patients and staff experiences of quality of forensic psychiatric care and this allows for internal and external comparisons [24, 25]. By comparing with other care deliverers, preferably those representing best known practice, participants can evaluate their own efforts and improvements over time [26].

The aim of the present study was to describe and compare patient and staff experiences of quality of care in two forensic psychiatric clinics over a period of 4 years.

## Methods

### Setting

Two forensic clinics, one in each county, were selected for comparison as they were highly similar in a range of aspects; both were the single forensic clinic serving a county with about 280,000 inhabitants each. The clinics were of about the same size and organization, about 30 bed-sites and a staff of about 100, psychiatrists and forensic nurses included. Both clinics had a medium security classification, receiving patients sentenced to forensic care with a multitude of psychiatric diagnosis. In spite of the similarities in prerequisites and organization, the two clinics had different reputations concerning the quality of their care; one with a reputation of offering high quality care, hereafter named Clinic B, and one with a reputation of quality shortcomings in their care, hereafter named Clinic A. When the management in clinic A decided to launch a program for quality improvement they invited the research team to follow the process by having yearly surveys of staff and patient opinions of the quality of the care.

During the 4 years of study (2011–2014), there were some changes which shortly will be described. In the end of 2012, it was decided that Clinic A should be relocated to new facilities adjusted to forensic psychiatric care, especially in terms of safety and security. The following year the staff prepared for moving the clinic and in December 2013, the clinic moved to an environment which was new for both patients and staff. In 2014, Clinic B was merged with another psychiatric clinic which led to changes in management organization.

### Participants

#### Patients

All patients in ongoing forensic care meeting the following inclusion criteria during the time of data collection were invited to participate: (a) being able to understand and read Swedish and (b) being cognitively able to answer the questionnaire. All of the patients were involuntarily admitted according to the Forensic Mental Care Act (1991:1129). This law ensures that people who have committed a crime, which normally would lead to prison, are transferred to forensic psychiatric care because of a serious mental disorder. In Sweden, the care is not time-specific but certain criteria must be fulfilled before discharge; there must be a low risk for re-offending, the patient must have a permanent home and structured days with regular activities. In both clinics, the mean length of treatment was 55 months. Both men and women were treated at the wards and about 15–20% of the patients were women, which is equivalent with national proportions. The most common diagnoses among the patients in both clinics were schizophrenia and other psychotic disorders [27]. More characteristics of the participating patients are presented in Table 1.

**Table 1:** Characteristics of the study groups—patients (percentages on answered questionnaires are based on the number of inpatients at the clinics when the questionnaires were distributed. All other percentages are based on those who answered the questionnaires)

Clinic	Variable	Value	Year			
			2011	2012	2013	2014
			n (%)	n (%)	n (%)	n (%)
A	Inpatients		25	23	26	21
	Answered questionnaires		11 (44)	13 (57)	15 (58)	9 (43)
B	Inpatients		34	33	32	33
	Answered questionnaires		13 (38)	15 (45)	18 (56)	11 (39)
A	Gender	Female	3 (27)	1 (8)	2 (13)	1 (11)
		Male	8 (73)	12 (92)	13 (87)	8 (89)
B	Gender	Female	2 (15)	3 (20)	3 (17)	3 (27)
		Male	11 (85)	12 (80)	15(83)	8 (73)
A	Age	18–29	2 (18)	1 (8)	3 (20)	2 (22)
		30–39	4 (36)	5 (39)	4 (27)	4 (44)
		40–49	4 (36)	6 (46)	7 (47)	2 (22)
		50–59	1 (9)	1 (8)	0	1 (11)
		60–69	0	0	1 (7)	0
B	Age	18–29	3 (23)	5 (33)	4 (22)	3 (27)
		30–39	4 (31)	6 (40)	9 (50)	5 (46)
		40–49	4 (31)	2 (13)	1 (6)	1 (9)
		50–59	2 (15)	2 (13)	4 (22)	2 (18)
A	Educational level	Elementary school	5 (42)	4 (33)	5 (36)	3 (38)
		Upper/secondary school	7 (58)	6 (50)	7 (50)	5 (63)
		College/university	0	2 (17)	2 (14)	0
B	Educational level	Elementary school	4 (31)	5 (33)	2 (11)	7 (64)
		Upper/secondary school	7 (54)	9 (60)	14 (78)	4 (36)
		College/university	2 (15)	1 (7)	2 (11)	0

#### Staff

Healthcare staff members working at the same clinics as the patients were asked to participate in the study. The number of employees was around 100 in Clinic A and 110 in Clinic B during the time of the study. According to management, there was a stability among the staff during the time of the study, which means that most of them were employed and were able to participate in the study every year. Among those answering the questionnaires in the two clinics, in total over the 4 years, 21.9% were forensic nurses, 65.1% were nursing assistants and 4.8% were counsellors. Doctors, psychologists, social workers and occupational therapists, were 8.2%. A majority (89%) of the staff members had worked for more than 3 years. Most worked day shifts only (69%), 12% worked night shifts only and 19% worked both day and night. More characteristics of the staff are presented in Table 2.

**Table 2:** Characteristics of the study groups—staff (percentages are based on those who answered the questionnaires)

Clinic	Variable	Value	Year			
			2011	2012	2013	2014
			n (%)	n (%)	n (%)	n (%)
A	Answered questionaires		86	88	82	71
B	Answered questionaires		76	81	62	52
A	Gender	Female	38 (44)	36 (41)	36 (44)	28 (39)
		Male	48 (56)	52 (59)	46 (56)	43 (61)
B	Gender	Female	46 (61)	47 (58)	37 (59)	30 (58)
		Male	30 (39)	34 (42)	26 (41)	22 (42)
A	Age	18–29	6 (7)	9 (10)	7 (8)	6 (9)
		30–39	13 (15)	11 (13)	11 (13)	12 (17)
		40–49	15 (17)	20 (23)	20 (24)	12 (17)
		50–59	23 (27)	25 (28)	26 (33)	28 (39)
		60–69	29 (34)	23 (26)	18 (22)	13 (18)
B	Age	18–29	5 (7)	6 (7)	6 (10)	9 (17)
		30–39	20 (26)	18 (22)	15 (24)	10 (19)
		40–49	25 (33)	32 (40)	20 (32)	16 (31)
		50–59	20 (26)	20 (25)	15 (24)	11 (21)
		60–69	6 (8)	5 (6)	6 (10)	6 (12)

### Procedure

In October each year (2011–2014), patients and staff, according to selection criteria were asked to rate their experiences of the quality of care. The first measure was conducted before the program for quality improvement was implemented in Clinic A and could count as a baseline. Before the study started, patients and staff were informed orally and in writing about the aim and design of the study. This information was given by contact persons at the clinics according to instructions from the researchers. Individuals who were interested in participating in the study were asked to respond anonymously and to return the completed questionnaire in a sealed envelope. The patients were also informed that participation would not have any effect on the care provided. This procedure was repeated each of the 4 years. The exact response rate is not known because the lists provided for registration of this were not complete. In total, 105 questionnaires were answered by patients and 598 by staff (Tables 1 and 2).

### Measurement

The instruments QPC-FIP [18] and the QPC-FIPS [19] were used for data collection. QPC-FIP and QPC-FIPS have the same number of items (n = 34) and are similar in content but the wording in QPC-FIP is from the patient perspective and in QPC-FIPS from the staff perspective. The instruments are self-administered and are designed to measure seven dimensions of quality of care. The dimensions include (1) “Encounter” (eight items, e.g. “staff has time to listen to me” or “the staff has time to listen to the patients”), (2) “Participation” (eight items, e.g. “I receive information about treatment alternatives” or “the patients receive information about treatment alternatives”), (3) “Discharge” (three items, e.g. “I receive information about where I can go if I need help following discharge” or “the patients receive information about where they can go if they need help following discharge”), (4) “Support” (four items, e.g. “the staff help me to understand that it is not shameful to suffer from mental health problems” or “the staff help the patients to understand that it is not shameful to suffer from mental health problems”), (5) “Secluded environment” (two items, e.g. “I have access to a place that is private where I can withdraw when I want to be left in peace and quiet” or “the patients have access to a place that is private where they can withdraw when they want to be left in peace and quiet”), (6) “Secure environment” (three items, e.g. “I feel secure with fellow patients” or “The patients feel secure with their fellow patients”) and (7) “Forensic specific” (six items, e.g. “I am informed of my rights” or “The patients are informed of their rights”). All items are scored on 4-point forced choice Likert scales, ranging from 1 (totally disagree) to 4 (totally agree). For all items, it is possible to respond with “not applicable”. Both instruments have background questions covering the demographic variables of the respondent.

### Data analysis

All statistical analyses were conducted using SPSS 22. Before analysis, questionnaires with 30% or more missing items were discarded. Imputation was performed by replacing missing data points with the overall mean of that item in questionnaires having less than 30% missing items. Non-parametric statistics were performed on the QPC subscale scores and background variables. The Mann–Whitney U test was used to test for group differences as well as differences across time within groups and clinics, since it was not possible to track individuals across years. A *p* value less than 0.05 was regarded as statistically significant.

## Results

### Analysis of background variables in QPC-FIP and QPC-FIPS

There were few differences between the patients and the staff at the two clinics. In 2011 fewer patients at Clinic A than in Clinic B knew who their responsible doctor was (χ^2^ = 6.19, p = 0.039, Fishers exact test) and where to complain if not satisfied with the provided care (χ^2^ = 5.85, p = 0.041, Fishers exact test). There were no differences in any other background variable between patients at the two clinics in year 2012, 2013 or 2014. In regard to the staff, some differences between the clinics were found for separate years, such as in 2011 staff at Clinic A reported to have less time to perform their tasks compared to staff in Clinic B (p < 0.010) and in 2013 the staff at Clinic A perceived their work more professionally enriching compared to staff in Clinic B (p = 0.034).

The rest of the result is presented in three parts. First, we compared patients’ and staff perceptions of quality of care from year to year within Clinic A and within Clinic B. Secondly; we compared the ratings between patients and staff in Clinic A and Clinic B respectively. Thirdly, we compared patient and staff ratings between the clinics. QPC-FIP data per dimension for the patients are shown in Table 3 and QPC-FIPS data for the staff in Table 4.

**Table 3:** Patients’ evaluation of quality of psychiatric forensic care—per dimension in QPC-FIP. Mean values 2011–2014 (1 = totally disagree and 4 = totally agree)

QPC	2011	2012	2013	2014
Clinic A				
Encounter	2.89 (0.69)	3.27 (0.87)	2.64 (0.71)	2.98 (0.80)
Participation	2.47 (0.61)	2.90 (0.88)	2.33 (0.80)	2.21 (0.77)
Discharge	2.65 (0.72)	3.18 (1.14)	2.64 (0.61)	2.56 (1.22)
Support	2.44 (1.42)	3.08 (1.01)	2.49 (0.42)	2.75 (1.16)
Secluded environment	3.64 (0.71)	3.46 (0.75)	3.42 (0.85)	3.44 (0.73)
Secure environment	2.64 (0.67)	3.31 (0.93)	2.55 (0.87)	2.81 (0.71)
Forensic specific	2.75 (0.52)	3.16 (0.88)	2.58 (0.65)	2.43 (0.80)
Clinic B				
Encounter	3.02 (0.90)	3.01 (0.94)	3.09 (0.72)	3.22 (0.81)
Participation	2.73 (0.92)	2.59 (0.95)	2.64 (0.72)	2.92 (0.49)
Discharge	2.85 (1.02)	2.98 (0.89)	2.88 (0.74)	3.18 (0.63)
Support	2.66 (1.08)	3.01 (0.79)	2.55 (0.57)	3.23 (0.52)
Secluded environment	3.67 (0.47)	3.42 (0.67)	3.58 (0.60)	3.45 (0.96)
Secure environment	3.00 (0.82)	2.86 (0.83)	3.15 (0.64)	2.97 (0.75)
Forensic specific	3.01 (0.96)	2.95 (0.84)	2.93 (0.60)	3.44 (0.60)

**Table 4:** Staff evaluation of quality of psychiatric forensic care—per dimension in QPC-FIPS. Mean values 2011–2014 (1 = totally disagree and 4 = totally agree)

QPC	2011	2012	2013	2014
Clinic A				
Encounter	3.33 (0.57)	3.33 (0.51)	3.44 (0.50)	3.45 (0.46)
Participation	2.70 (0.52)	2.74 (0.52)	2.89 (0.53)	2.93 (0.55)
Discharge	3.24 (0.58)	3.24 (0.58)	3.46 (0.57)	3.38 (0.55)
Support	3.40 (0.55)	3.44 (0.52)	3.56 (0.46)	3.50 (0.49)
Secluded environment	3.65 (0.55)	3.67 (0.58)	3.72 (0.45)	3.94 (0.16)
Secure environment	2.65 (0.51)	2.75 (0.51)	2.76 (0.52)	3.02 (0.54)
Forensic specific	3.28 (0.49)	3.32 (0.45)	3.40 (0.51)	3.39 (0.42)
Clinic B				
Encounter	3.23 (0.49)	3.24 (0.48)	3.41 (0.52)	3.39 (0.49)
Participation	2.76 (0.52)	2.80 (0.50)	2.98 (0.52)	2.69 (0.51)
Discharge	3.19 (0.56)	3.22 (0.63)	3.33 (0.55)	3.10 (0.66)
Support	3.29 (0.58)	3.33 (0.54)	3.45 (0.53)	3.30 (0.54)
Secluded environment	3.75 (0.38)	3.82 (0.36)	3.80 (0.42)	3.77 (0.42)
Secure environment	2.48 (0.59)	2.65 (0.55)	2.80 (0.56)	2.48 (0.45)
Forensic specific	3.27 (0.48)	3.28 (0.44)	3.35 (0.45)	3.20 (0.48)

### Patient and staff experiences of quality of care from year to year 2011–2014

#### Clinic A

##### Patient experiences of quality of care

In 2011 patients at Clinic A rated the quality of Secluded environment highest and Support as the lowest. At 2012, after 1 year with improvement work, ratings increased for all dimensions of quality of care, though only the increase in ratings of Secure environment was significant (U = 36.5, p = 0.038). In 2013 the ratings of the subscales Encounter (U = 52, p = 0.035), Discharge (U = 49.5 p = 0.025), and Secure environment (U = 48.5 p = 0.022) decreased compared with the 2012 ratings. In 2014, there were no significant differences in any subscale compared with 2013.

##### Staff experiences of quality of care

Staff also perceived that the quality of Secluded environment was highest in 2011 but they rated the quality of Secure environment as the lowest. In 2012, the ratings were similar with baseline. However, the staff rated that the quality of Participation (U = 3010, p = 0.027) and Discharge (U = 2839, p = 0.008) had increased in 2013; and that the quality of Secluded environment (U = 2197, p = 0.000) and Secure environment increased between 2013 and 2014 (U = 2060, p = 0.001).

#### Clinic B

##### Patient experiences of quality of care

In clinic B, there were minor changes in ratings among the patients during the years. The patients perceived that quality of Support decreased (U = 66, p = 0.021) between 2012 and 2013 and increased (U = 31, p = 0.004) between 2013 and 2014. Also in 2014, the quality of Forensic specific had increased from the previous year (U = 52, p = 0.032).

##### Staff experiences of quality of care

Compared to baseline, the quality of Secure environment improved in 2012 (U = 2589, p = 0.034) and the quality continued to improve the following year with significance in Encounter (U = 2121, p = 0.015) and Participation (U = 2222, p = 0.042). However, in 2014 staff perceived that the quality of Participation (U = 1293, p = 0.002) and Secure environment (U = 1217, p = < 0.001) had decreased.

### Differences between how patient and staff rated their experience of quality of care 2011–2014

#### Clinic A

In 2011, staff at Clinic A reported a higher quality than the patients in the dimensions Encounter (U = 292, p = 0.032), Discharge (U = 263, p = 0.013) and Forensic specific (U = 218, p = 0.003). In 2012, however, all these differences were gone, and the patients even reported a higher quality than the staff in relation to Secure environment (U = 301, p = 0.004). In 2013, the differences between staff and patient reported at baseline had re-emerged and again staff reported a higher quality than the patients in Encounter (U = 231, p = < 0.001), Discharge (U = 170, p = < 0.001), and Forensic specific (U = 200, p = < 0.001) but also in Participation (U = 349, p = 0.007) and Support (U = 71, p = < 0.001). In 2014 the differences continued and compared with the patients, staff reported a higher quality in Participation (U = 144, p = 0.007), Secluded environment (U = 197, p = 0.003) and Forensic-specific (U = 96, p = 0.001).

#### Clinic B

At baseline, the staff rated the quality of care higher than the patients in relation Support (U = 338, p = 0.046). However, in the same year the patients rated the quality of Secure environment higher than the staff (U = 327, p = 0.034). In 2012 there was only one significant difference where staff rated the quality of Secluded environment higher than patients (U = 395, p = 0.007). In 2013, staff perceived the quality higher than patients in relation to Discharge (U = 386, p = 0.015) and Support (U = 151, p = < 0.001) while patients rated the quality of Secure environment higher than staff (U = 394, p = 0.019). In 2014 there were no significance differences between staff and patient ratings of the quality of care.

### Comparisons between Clinic A and Clinic B 2011–2014

#### Between patients

**Fig. 1:**
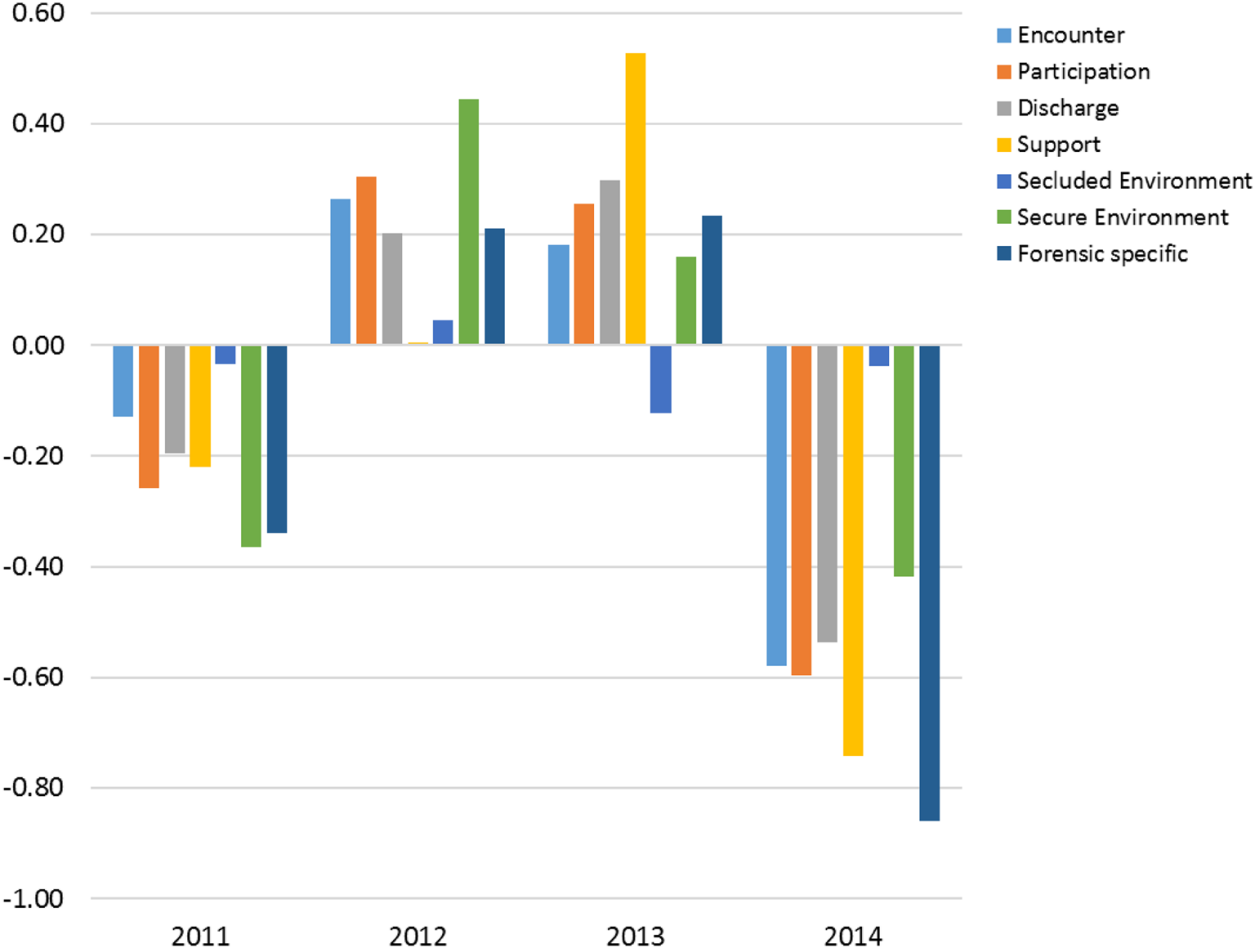
Differences in perceived quality of care between patients at Clinic A and Clinic B in QPC-FIP. A positive difference indicates greater perceived quality of care in Clinic A than in Clinic B. Negative differences indicate the opposite

Comparisons between patients in Clinic A and Clinic B showed no significant differences in reported quality of care at the baseline in 2011 or 2012. In 2013 however, the patients in Clinic A reported lower quality than the patients in Clinic B in relation to Secure environment (U = 74.5, p = 0.027). In 2014, the patients in Clinic A reported the quality of Participation and Forensic-specific aspects lower than the patients in Clinic B (U = 21, p = 0.013 and U = 16, p = 0.010, respectively). Figure 1 illustrates the differences in all dimensions.

#### Between staff

**Fig. 2:**
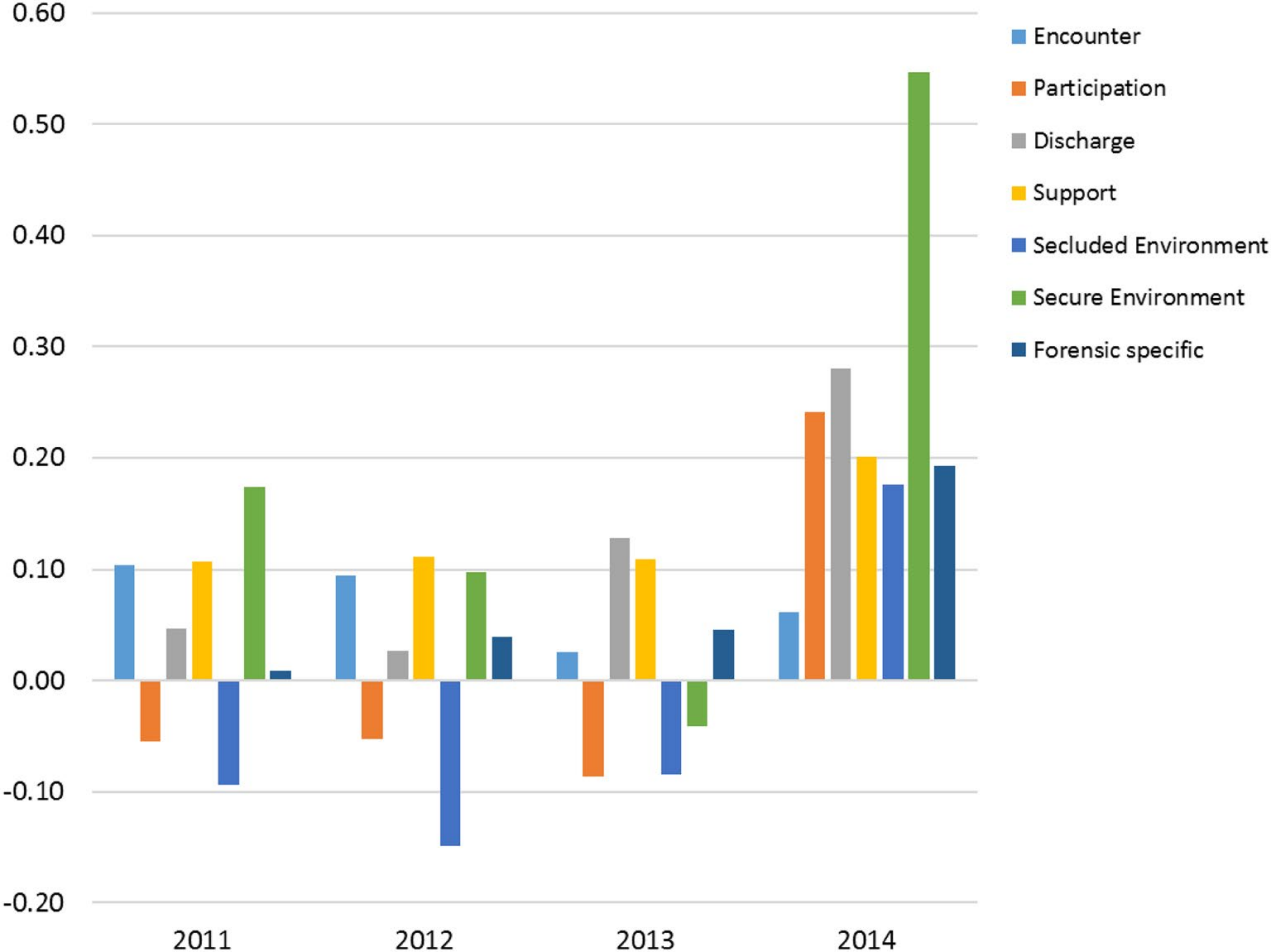
Differences in perceived quality of care between staff at Clinic A and Clinic B in QPC-FIPS. A positive difference indicates greater perceived quality of care in Clinic A than in Clinic B. Negative differences indicate the opposite

At baseline, the staff in Clinic A reported a higher quality than Clinic B in relation to Secure environment (U = 2866, p = 0.047) and then there were no differences between the clinics in 2012 and 2013. In 2014, however, as seen in Fig. 2, the staff in Clinic A reported a higher quality than Clinic B in relation to Participation (U = 1484, p = 0.014), Discharge (U = 1513, p = 0.019), Support (U = 1545, p = 0.029), Secluded environment (U = 1584, p = 0.005), Secure environment (U = 834, p = < 0.001) and Forensic-specific (U = 1547, p = 0.031).

## Discussion

The aim of the present study was to describe and compare patient and staff experiences of quality of care in two forensic psychiatric clinics over a period of 4 years. The repeated measurements showed that quality of care, as described by the patients, varied over time, with significant changes over the 4 years. The staff evaluations of the quality of care were more stable over time in both clinics compared with the patients. Generally, the staff rated the quality as being better than the patients did, a result that is similar to other studies [15], which shows the necessity to evaluate quality of forensic psychiatric care from both staff and patient perspectives.

In clinic A, improvement work started after the first measure in 2011. In spite of this, no sustained increase in quality of care was observed. However, on a group level there was stability of ratings among the staff during the 4 years in Clinic A, whereas more variation was found among the patients. The trend was an initial improvement from 2011 to 2012, followed by a negative change 2013 and 2014. This suggests that the patients are more sensitive to how the care is performed than staff, which can perhaps be expected; i.e. the patient in forensic psychiatric care is vulnerable because of mental illness and the fact that the patient actually lives in the environment during most of the treatment time [28, 29].

There was a negative trend among patients in clinic A after the improvement work in 2012. For example, there was a decrease in experienced quality in the dimensions of Encounter, Participation, Discharge, Support and Forensic-specific. It is interesting that staff reported an improved quality in relation to participation and discharge during the same period. It is possible that the coming move made the patients worried or that staff focused less on improvement work than in the previous year due to the preparations, without being aware of it. A similar study where staff only, not patients, answered a patient-centered care questionnaire before a relocation to new health care environments within forensic psychiatry and which included three follow-ups after the move, showed a sustainability of a person-centered ward atmosphere [30]. Work structures are however, together with professions and working relationships, the main factors which influence how workers engage in healthcare quality improvement work, and work structures tend to prevent their engagement [31]. It is also possible that that patients are more sensitive to changes than staff, or at least reacts to them more quickly.

A difference between staff and patients’ experiences of the quality of care is perhaps to be expected in coercive care. At baseline in Clinic A, group differences of quality in the dimensions of Encounter, Discharge and Forensic-specific were found and in all of them the staff rated the quality higher than the patients. The same pattern was found in an earlier study from forensic psychiatric care in Sweden [15]. Perhaps staff and patients have different viewpoints, due to different expectations, experience, knowledge and/or educational level [13, 15]. It might also be a consequence of the fact that the care is coercive. From a patient perspective, the care could be perceived as paternalistic or that staff misuse their power position; for example, with a bad encounter [28, 29, 32]. It is therefore possible that staff, based on their professional competence and experience, treat the patient with what they perceive to be high quality care and best-known practice, but it is not necessarily perceived as such by the patient [29]. It is important though, in all situations, to identify and reflect upon why differences exist.

There was a relative low number of participating patients. This could be explained by the fact that they are involuntarily admitted which combined with psychiatric disorders, might affect their ability and motivation to answer a questionnaire. An incidental finding in the study was that patient participation was rated as the lowest or second lowest of all dimensions among both patients and staff in all 4 years. There have been similar findings in earlier studies in forensic psychiatric care in Denmark and Sweden, which indicate that the concept of patient participation should be investigated further in this context [15, 20, 21, 33].

### Strengths and limitations

The design which employed yearly sampling over a period of 4 years and having participants consisting of both patients and staff from the same wards was a strength in this study. We also used an instrument validated for measuring quality of forensic psychiatric care, which was deemed to be relevant and sensitive to changes during improvement work. A limitation of the study is that we did not follow individuals over time even if we know there was stability among both the patients and the staff over the years. This makes the statistical power in the study lower, and it is possible that we did not find existing differences. There is also a risk of recall bias because of the long time between data collections and because the participants did not keep a diary or journal throughout each year. Therefore, the results should be interpreted with caution.

## Conclusions

People working in healthcare have a responsibility of not only accomplishing their daily commitments, but also to improve and develop care systems, based on evidence and experience [34]. Forensic psychiatric care occurs in a context where there are challenges for the staff when developing a care-promoting climate of high quality because the care is coercive and patients are often treated against their will. By highlighting what staff and patients perceive as both high and low quality of care, future interventions can be planned with good precision and improvement work can be evaluated quickly. The results also indicate the value of considering both patients’ and staff experiences during organizational changes. With regular measurements and sufficient resources, training, support and leadership, the chances of successful improvement work increase [35]. Such knowledge is important in forensic nursing practice, for teaching and for management and for decision makers in the constant work of improving forensic psychiatric care.
